# The Global Leadership Initiative on Malnutrition (GLIM) Tool for Nutritional Assessment of Adult Patients After Sleeve Gastrectomy: Is It the Recommended Tool?

**DOI:** 10.3390/nu17061074

**Published:** 2025-03-19

**Authors:** Amani N. Alotaibi, Fahad Bamehriz, Nadia A. Aljomah, Khalid Almutairi, Shabana Tharkar, May Al-Muammar, Adel Alhamdan, Dara Aldisi, Mahmoud M. A. Abulmeaty

**Affiliations:** 1 Department of Community Health Sciences, College of Applied Medical Sciences, King Saud University, Riyadh 11362, Saudi Arabia; 444204280@student.ksu.edu.sa (A.N.A.); kalmutairim@ksu.edu.sa (K.A.); stharkar@ksu.edu.sa (S.T.); malmuammar@ksu.edu.sa (M.A.-M.); adel@ksu.edu.sa (A.A.); daldisi@ksu.edu.sa (D.A.); 2 Surgery Department, Upper GI Surgery, King Khalid University Hospital, King Saud University, Riyadh 12372, Saudi Arabia; fbamehriz@ksu.edu.sa (F.B.); nadia.aljomah@gmail.com (N.A.A.)

**Keywords:** global leadership initiative on malnutrition (GLIM), malnutrition, sleeve gastrectomy, subjective global assessment, validity

## Abstract

**Background/Objectives**: Malnutrition frequently occurs following bariatric surgery and can lead to higher morbidity rates, hospitalizations, and extended hospital stays. Nutritional assessment tools such as the Global Leadership Initiative on Malnutrition (GLIM) are not validated for diagnosis of malnutrition following bariatric surgery. This study aimed to assess the validity of GLIM criteria in evaluating the nutritional status of post-sleeve gastrectomy patients compared to the Subjective Global Assessment (SGA). **Methods**: A total of 47 adult patients who underwent sleeve gastrectomy (SG) from 6 months to 2 years prior were evaluated using the GLIM and SGA. Additionally, multiple pass 24 h recall was collected for two days, and macronutrient analyses were conducted using ESHA software (version 11.11.x). Agreement between both tools was determined using Kappa (κ) statistics, and the Receiver Operating Characteristics (ROC) curve was used to establish sensitivity and specificity. **Results**: The study found that malnutrition was diagnosed in 48.9% and 42.6% of patients according to the GLIM and SGA criteria, respectively. The GLIM criteria exhibited inadequate accuracy (AUC = 0.533; 95% CI, 0.38–0.72) with a sensitivity and specificity of 55.0% and 55.6%, respectively. The agreement between both tools was determined to be poor (κ = 0.104). **Conclusions**: GLIM did not show sufficient agreement with SGA. Consequently, the criteria of GLIM may need revision for better diagnosis of malnutrition in post-sleeve gastrectomy patients.

## 1. Introduction

With the burgeoning rise in obesity worldwide, bariatric surgeries are considered to be effective procedures for weight reduction [1,2]. Bariatric surgeries are usually recommended as a weight-losing procedure for individuals with a Body Mass Index (BMI) > 35 kg/m^2^, independent of the presence or absence or severity of co-morbidities. However, bariatric surgeries have been associated with adverse effects like gastroesophageal reflux disease, dyspepsia, loss of appetite, and abdominal distension, leading to nutritional inadequacy and the risk of developing consequent malnourishment post-surgery [3,4]. Malnutrition in the form of protein-energy malnutrition and micronutrient deficiencies is one of the most common postoperative nutritional complications [5]. Nutritional guidelines for bariatric surgery patients mandate detailed pre-operative and regular post-operative nutritional assessment [6]. Subjective Global Assessment (SGA) is a nutrition assessment tool considered a gold standard in diagnosing malnutrition. It includes a comprehensive evaluation of patient history, physical examination, and structured assessment of clinical parameters to diagnose malnutrition [7]. However, validity and reliability reviews suggest using more than one assessment tool, as none of the existing tools are reliable in determining malnutrition in different clinical settings with a range of patient conditions [8,9].

The Global Leadership Initiative on Malnutrition (GLIM) was developed in 2018 with the help of multiple scientific societies and attempted to revolutionize and standardize the diagnosis of malnutrition in various clinical settings by introducing GLIM criteria [10]. Due to novelty since its inception, the consensus recommends that scientific researchers conduct studies to validate the tool in different patient populations and clinical settings [11].

The GLIM criteria involve a two-step approach: first, risk screening to identify patients at risk of malnutrition using any validated screening tool, such as NRS-2002, and second, the actual diagnosis of malnutrition using etiologic and phenotypic components. While many studies have evaluated GLIM criteria in chronically ill patients, hospitalized patients, and those with cancer and diabetes, no studies have assessed the validity of GLIM criteria in diagnosing malnutrition in patients who have undergone sleeve gastrectomy surgery (SG) [12,13,14]. Thus, the primary objective of the present study is to validate the GLIM criteria as a tool for diagnosing malnutrition during nutritional assessment in post-SG adult patients in Saudi Arabia, using SGA as a reference. The secondary aim is to determine whether there is a need for GLIM criteria revision to diagnose malnutrition in adult patients with post-SG.

## 2. Materials and Methods

### 2.1. Study Design

This cross-sectional study included patients who underwent sleeve gastrectomy and visited the bariatric surgery multidisciplinary clinic of the King Khalid University Hospital between September 2023 and May 2024. Study participants were recruited consecutively from the attendants for the post-operative follow-up visits. The patient registry and appointment list were reviewed first to determine the eligibility of each possible participant. The eligibility criteria included adult patients (18–65 years) who underwent sleeve gastrectomy 6 to 24 months ago and from various socioeconomic and demographic backgrounds. Patients with malignancy, debilitating diseases that rapidly deteriorate muscle mass or neurological status (such as muscular dystrophy, cerebral palsy, cystic fibrosis, multiple sclerosis, etc.), pregnant and lactating women, and those aged less than 18 and above 65 were excluded.

The sample size required for the study was calculated using Med-Calc Statistical Software (version 23.1.7) based on the area under the ROC curve, considering SGA as the gold standard to determine the validity of GLIM. With an alpha level of 0.05, beta level of 0.10 (90% power), a minimum expected Area under the Curve (AUC) of 0.877, a null hypothesis value of 0.5, and a ratio of well-nourished to malnourished of 5.31 (85% well-nourished and 16% malnourished), based on the previous study conducted by Albukhari et al., the required number of study subjects was 38 participants.

### 2.2. Data Collection

Following an explanation of the study details and purpose, patients were approached for their consent and participation. The searchers collected the data using a face-to-face interview method. A well-structured data collection sheet included sections for demographic information to be collected and anthropometric measurements, bioelectrical impedance analysis, and blood tests were conducted. Detailed multiple pass 24 h recalls were conducted for 2 days, followed by SGA assessments.

#### 2.2.1. Demographic Data

Socio-demographic characteristics included age, gender, marital status, education level, previous medical history, medication, smoking, gastrointestinal symptoms, sleeping hours, and use of nutrition supplements.

#### 2.2.2. Anthropometric Measurement

Anthropometric parameters, including body weight and height, were measured, and Body Mass Index (BMI) was calculated as weight in kilograms divided by the square of height in meters (kg/m^2^). The percentage of weight loss (WL%) was calculated by dividing the weight lost by the patient by the previous (preoperative) weight and multiplying it by 100.

#### 2.2.3. Body Composition Analysis

Body composition was determined by Bioelectrical Impedance Analysis (BIA) using the TANITA BC-418 MA (Tanita Corp., Tokyo, Japan) and was measured as fat mass, fat-free mass, muscle mass, and fat percentage. The study subjects were instructed to cleanse and wipe their feet and hands with electrolyte tissue wipes to eliminate the potential impediments that could interfere with the electrical current. Participants were also instructed to remove transmitters such as smartwatches and mobile phones. The patient’s muscle mass was assessed based on the Appendicular Skeletal Muscle Mass Index (ASMMI) expressed in ASMM/height^2^ (Kg/m^2^), where ASMM is the sum of the measured muscle mass of the four limbs. For the Saudi adults, it was reported that the median skeletal mass was 7.7 kg/m^2^ in women and 10.7 kg/m^2^ in men [15].

#### 2.2.4. Biochemical Assessment

Nutrition-related laboratory tests, including complete blood count, protein level, hemoglobin, glucose, and lipid status, were performed after a 12 h fast. Serum albumin, C-reactive protein (CRP), and neutrophil-to-lymphocyte ratio (NLR) measurements were taken from recent electronic medical records (within 1 week). The neutrophil-to-lymphocyte ratio (NLR) was calculated by dividing the neutrophil count by the lymphocyte count.

#### 2.2.5. Dietary Assessment

A dietary assessment was performed by using multiple-pass 24-h diet recall for two days, one on a weekday and one on a weekend. The data were analyzed using ESHA food processor software version 11.11 (Esha Research Inc, Salem, OR, USA) to measure the macronutrient and micronutrient content in the patient’s diet [16]. A survey on the commitment to the administration of nutritional supplements such as bariatric surgery-specific multivitamins, including iron, calcium, vitamin D, and B12, was conducted.

#### 2.2.6. Subjective Global Assessment (SGA)

The components of SGA involve the history of self-reported weight changes over the past six months, dietary intake modifications, gastrointestinal symptoms persisting for more than two weeks, functional capacity, nutritional needs related to the underlying disease, and physical examination for signs such as loss of subcutaneous fat, muscle wasting, and edema or ascites. These criteria are used to classify patients into three categories: A (well-nourished), B (moderately malnourished), or C (severely malnourished). Patients falling into categories B or C are considered to be malnourished [17].

#### 2.2.7. Nutrition Risk Screening 2002 (NRS-2002)

The NRS-2002 is a two-step screening tool. In the first step, patients are assessed for malnutrition risk based on specific criteria such as low BMI (less than 20 kg/m^2^), weight loss, reduced dietary intake, and severity of illness. If any of these criteria are met, the second step involves further assessing nutritional parameters and grading disease severity. Patients are then categorized using a scoring system, with a score of 3 or higher indicating nutritional risk [18]. 

#### 2.2.8. GLIM Criteria

After initial screening using the NRS-2002, the GLIM tool was applied. The GLIM criteria consist of two components: etiologic criteria (including reduced food intake or assimilation and disease burden/inflammation) and phenotypic criteria (weight loss, low BMI, and reduced muscle mass). A diagnosis of malnutrition is made when at least one component from each category is met, and the severity is assessed using phenotypic threshold levels. This study combines patients with moderate (stage 1) or severe (stage 2) malnutrition. The phenotypic criteria are considered positive if there is a weight loss of more than 5% over the previous six months or more than 10% over six months. Asian people with a BMI below 18.5 kg/m^2^ or reduced muscle mass (median of 7.7 kg/m^2^ in women and 10.7 kg/m^2^ in men) satisfy the GLIM criteria for malnutrition risk. The etiologic criteria are met with serum albumin levels below 35 g/L or a NLR (neutrophil-to-lymphocyte ratio) equal to or greater than 3 or less than 0.7, indicating inflammation or disease severity [19]. Additionally, the GLIM criteria suggest evaluating inflammation based on acute disease/injury or chronic disease-related factors if laboratory data are unavailable [20]. Self-reported percentages of actual food intake (0%, 25%, 50%, 75%, and 100%) compared to usual intake were used to measure reduced food intake or assimilation. Additionally, gastrointestinal symptoms like diarrhea and vomiting or any clinical manifestations impacting food assimilation were evaluated.

### 2.3. Statistical Analysis

The data analysis was conducted using IBM SPSS Statistics for Windows, Version 26.0 (SPSS, version 25, Chicago, IL, USA). The normality of the parameters was determined using the Shapiro–Wilk test. Continuous variables were presented as Mean ± standard deviation (SD) for normally distributed data and Median and interquartile range (IQR) for skewed data. Categorical variables were expressed as numbers (*n*) and percentages (%).

The chi-square test of proportion was employed to analyze categorical variables. In addition, Student’s *t*-test and the Mann–Whitney U test were utilized to perform inferential statistics comparing continuous data between groups with and without malnutrition.

To establish the concurrent validity of the GLIM criteria, the area under the curve (AUC) of the Receiver Operating Characteristics (ROC), along with a 95% confidence interval (CI), sensitivity, and specificity, were used, with SGA serving as the gold standard. When evaluating the accuracy according to the ROC, an AUC greater than 0.90 was considered outstanding, 0.80 to 0.90 denoted very good accuracy, 0.70 to 0.80 indicated good accuracy, 0.60 to 0.70 signified poor accuracy, and 0.50 to 0.60 represented failed accuracy [21]. The confusion matrix of actual malnutrition diagnosed with SGA and predicted malnutrition by GLIM was conducted. Accuracy, misclassification rate, sensitivity, specificity, and precision were calculated for the GLIM tool.

## 3. Results

**Figure 1:**
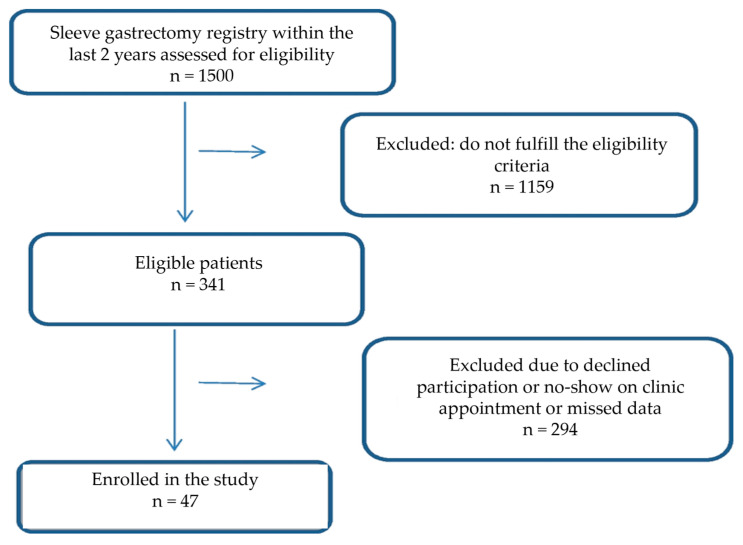
Flowchart of study participants.

A total of 47 patients (18 males and 29 females) were enrolled in this study (Figure 1). The median age of participants was 35.0 ± 22 years. Most of the patients were literate and single. Around 69.6% had the surgery within the last year, and 74.5% reported having other medical conditions. Most patients took multivitamins (72.3%), but few used protein supplements (12.8%). Based on the NRS-2002 criteria, 72.3% of patients were at risk of malnutrition. The demographic and nutritional characteristics of the study patients are shown in Table 1.

Table 2 detail the patients’ measurements, nutritional status, and malnutrition criteria using GLIM and SGA. According to GLIM and SGA criteria, the prevalence of malnutrition was 48.9% and 42.5%, respectively. Weight, BMI, and fat mass were significantly different between the healthy and malnourished groups according to both criteria. The fat percentage also showed a significant difference when assessed using the SGA criteria. Muscle mass was insignificantly different between the well-nourished and malnourished groups according to both criteria.

According to the two criteria, the levels of macro and micronutrients did not differ significantly between the well-nourished and malnourished groups (Table 3).

Additionally, the levels of Albumin, NLR, and CRP did not differ between the groups using either criterion, as shown in Table 4.

Table 5 provides information about the prevalence of phenotypic and etiologic criteria of malnourished patients according to the GLIM guidelines. In the phenotypic criteria, weight loss was observed in 48.9% of malnourished patients and reduced muscle mass in 100% of the malnourished patients (*p* < 0.0001). However, the etiological criteria showed no difference between the two groups in terms of reduced food intake (*p* = 0.885).

The receiver-operating characteristic (ROC) curve plots the true positive rate (sensitivity) against the false positive rate (1-specificity) at GLIM criteria compared with SGA.

**Figure 2:**
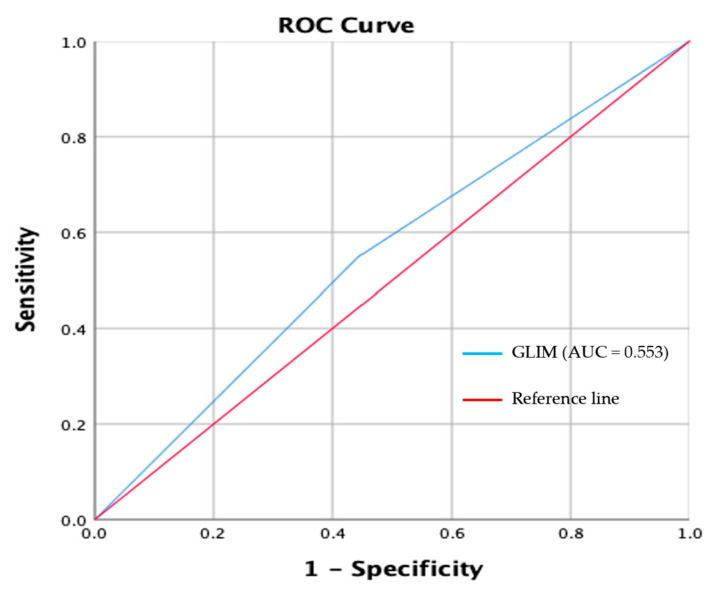
Receiver-operating characteristic (ROC) curve plot of the true positive rate (sensitivity) rate against the false positive rate (1-specificity) at GLIM criteria compared with SGA.

GLIM criteria showed poor agreement with SGA (κ = 0.10, *p* = 0.474). ROC analysis showed that GLIM criteria had a poor sensitivity (55.0%) and specificity (55.6%) compared with SGA. The AUC clearly showed the failed ability of the GLIM criteria to diagnose malnutrition when compared with SGA (AUC = 0.553; 95% CI, 0.38–0.72) (Figure 2).

The confusion matrix of actual malnutrition, as confirmed by SGA and predicted malnutrition by GLIM, is presented in Table 6. For GLIM, accuracy = 55.32%; misclassification rate = 44.68%; sensitivity = 57.14%; specificity = 55.55%; and precision = 47.82%.

Furthermore, we compared false positive (FP) and false negative (FN) groups (Table 7) to clarify further the reasons for the disagreement between GLIM and SGA. All the tested variables showed insignificant differences (*p* trend > 0.05), indicating that the disagreement is not due to the different characteristics of participants in both extremes.

## 4. Discussion

Patients often experience nutritional deficiencies after undergoing sleeve gastrectomy surgery. Early detection of malnutrition is crucial for maintaining critical health post-surgery. The GLIM consensus recently introduced a new two-step method for diagnosing malnutrition, and validation studies in various clinical settings are required [22]. This study, for the first time in the Arabic region, assesses the validity of GLIM criteria compared to SGA criteria in identifying malnutrition in post-sleeve gastrectomy patients. The primary goal of this study was to validate the use of GLIM criteria as a tool for diagnosing malnutrition in post-sleeve gastrectomy patients, using SGA criteria as the reference standard.

According to the GLIM criteria, the prevalence of malnutrition was 48.9%, while SGA data indicated a prevalence of 42.6%. This study revealed poor agreement (κ = 0.10, *p* = 0.474) between the GLIM and SGA criteria for diagnosing malnutrition in post-sleeve gastrectomy patients. The GLIM criteria were not found to be an accurate method for identifying malnutrition in post-sleeve gastrectomy patients, as indicated by their low sensitivity of 55.0%, specificity of 55.6%, and AUC of 0.553 when compared to SGA.

The accuracy of the GLIM criteria in diagnosing malnutrition has been evaluated in several studies with varying results. Allard et al. found good specificity (89.8%) but low sensitivity (61.3%) when using SGA as a comparator in Canadian hospital patients [14]. A study by Fontane et al. among Spanish patients reported 78% sensitivity and 86% specificity and did not recommend the use of GLIM due to sensitivity below 80% [13]. In contrast, a meta-analysis by Huo et al. reported high diagnostic accuracy of GLIM criteria with a sensitivity of 0.81, specificity of 0.80, and AUC of 0.87 when using SGA as a reference standard [23], while Brito et al., in a similar study, obtained sensitivity and specificity values of 87% and 82%, validating the GLIM criteria [24].

However, our study did not achieve concurrent validity due to low specificity and sensitivity. Our findings contradict some previous studies carried out on patients with other comorbidities in various settings. A recent study validating GLIM with SGA reference in type 2 diabetes patients found a good level of agreement with k = 0.778, a sensitivity of 77.8%, and an excellent specificity of 97.6% [12].

Furthermore, phenotypic criteria were met by almost all of the patients in the current study; those who lost weight by more than 10% after six months or by more than 5% in the six months before the study fall into this category. Furthermore, 70% of participants lost weight between six months and a year, which explains the rapid weight loss observed in the first half of the study. This weight loss is an indicator of SG success; however, GLIM considers it a sign of malnutrition. Alternative parameters such as reduced muscle mass and function or normalization of fat-to-muscle mass ratio should be sought in the future and should replace weight loss and low BMI. Reduced muscle mass is a better and more important phenotypic criterion indicator of malnourishment after weight loss surgery. The present study found that the group suffering from malnutrition exhibited notably reduced muscle mass (*p*-value < 0.0001). This outcome aligns with a study that assessed adult patients’ muscle mass by BIA at three, six, and twelve months following SG [25].

In terms of etiological criteria, the most prevalent criterion observed in patients following sleeve gastrectomy surgery was decreased food intake, which was consistent in both undernourished and well-nourished individuals. CRP, NLR, and hypoalbuminemia are commonly used as potent inflammatory markers. The GLIM group recommends low albumin as a marker of inflammation [10]. However, in the current study, albumin, as an inflammatory marker, did not show significant differences between the well-nourished and malnourished groups. Furthermore, patients with normal CRP levels (<3 mg/L) showed similar percentages in both GLIM-based well-nourished and malnourished groups. This normalization of inflammatory markers is expected in our sample due to the duration that lasted after surgery. It was reported that serum CRP levels drop after bariatric surgery in a manner that mimics and correlates with weight loss [26]. It was reported that CRP starts declining 1 month after surgery and may be normalized 6 months after sleeve gastrectomy [27].

Taken together, the use of improper tools may reduce the diagnostic and prognostic value. The GLIM tool is a promising malnutrition diagnostic tool that is validated for clinical use in many disease populations. However, patients in the field of metabolic and bariatric surgery (MBS) have a special case where clinicians find difficulty in differentiating between intended and unintended weight loss in the MBS population. We strongly suggest a modified version of GLIM that includes weight-free components such as muscle mass, muscle function, and fat-to-muscle mass ratio normalization.

The major limitations of the study include data from a single center, which could be addressed by conducting multicentric studies in the future. Additionally, the study’s cross-sectional design is another limitation.

## 5. Conclusions

In conclusion, this study found that 48.9% of patients met the criteria for malnutrition according to GLIM standards. It was also observed that the GLIM criteria provided a reliable framework for identifying malnutrition independently. However, the results indicated low sensitivity and specificity and were inconsistent with SGA assessments. This disparity could be explained by the fact that patients who were considered well-nourished by SGA were considered malnourished while using the GLIM tool.

Additionally, during the development of nutritional guidelines for post-bariatric surgery patients, it is advisable to use modified GLIM criteria based on phenotypic or etiological considerations for greater accuracy and relevance.

## Figures and Tables

**Table 1:** Demographic and nutritional characteristics of the post-sleeve gastrectomy patients.

Variable	N (%)
Age in years (Median ± IQR)	35.0 ± 22
Academic level	
Illiterate	2 (4.3)
Elementary	5 (10.6)
High school	17 (36.2)
University	21 (44.7)
Higher education	2 (4.3)
Marital Status	
Single	24 (51.1)
Married	17 (36.2)
Divorced	4 (8.5)
Widowed	2 (4.3)
Nutrition Supplement (Multivitamins, vitamin D and B12)	
Yes	34 (72.3)
No	13 (27.7)
Protein supplementation	
Yes	6 (12.8)
No	41 (87.2)
Duration post-sleeve gastrectomy	
≥6 months–1 year	32 (69.6)
1–1.5 years	5 (10.9)
1.5–2 years	9 (19.6)
Comorbidities Diseases	
Yes	13 (74.5)
No	12 (25.5)
Nutrition Status	
GLIM	
Well-nourished	24 (51.1)
Malnourished	23 (48.9)
SGA	
Well-nourished	27 (57.4)
Malnourished	20 (42.6)
NRS-2002	
At risk of malnutrition	34 (72.3)
Not at risk of malnutrition	13 (27.7)

**Table 2:** (**a**) Anthropometric status post-sleeve gastrectomy according to GLIM criteria. (**b**) Anthropometric status post-sleeve gastrectomy according to SGA.

**(a)**				
**Variable**	**GLIM**			
	**All** ***n* = 47**	**Well-Nourished** **(*n* = 24)**	**Malnutrition** **(*n* = 23)**	***p*** **Value**
Weight	82.28 ± 19.03	84.95 (32.5)	70.70 (25.1)	0.012
BMI	31.29 ± 5.98	32.45 (10.55)	27.60 (8.10)	0.042
Muscle mass	49.87 ± 11.67	51.94 ± 10.72	47.72 ± 12.45	0.221
Fat mass	28.92 ± 14.86	33.55 ± 14.89	24.09 ± 13.49	0.027
FFMI	20.11 ± 3.67	20.56 (3.15)	19.96 (4.41)	0.476
Fat percentage	3.40 ± 10.39	39.25 (16.85)	28.20 (17.8)	0.225
ASMMI	9.39 ± 3.28	9.01 (2.73)	8.94 (2.74)	0.125
**(b)**				
**Variable**	**SGA**			
	**All** ***n* = 47**	**Well-Nourished** **(*n* = 27)**	**Malnutrition** **(*n* = 20)**	***p*** **Value**
Weight	82.28 ± 19.03	85.30 (32.0)	71.15 (17.1)	0.031
BMI	31.29 ± 5.98	33.90 (9.70)	26.90 (7.45)	0.004
Muscle mass	49.87 ± 11.67	49.97 ± 13.03	49.75 ± 9.86	0.948
Fat mass	28.92 ± 14.86	33.78 ± 14.49	22.35 ± 12.97	0.007
FFMI	20.11 ± 3.67	20.61 (4.14)	19.74 (3.00)	0.212
Fat percentage	3.40 ± 10.39	38.90 (18.5)	32.25 (20.6)	0.013
ASMMI	9.39 ± 3.29	9.06 (2.32)	8.95 (1.98)	0.983

Data are presented as mean ± standard deviation (SD) or median and (IQR) and analyzed by *t*-test or Mann–Whitney U test. *p*-value ≤ 0.05 is considered statistically significant. Body Mass Index (BMI), Appendicular Skeletal Muscle Mass Index (ASMMI), and Fat-Free Muscle Mass Index (FFMI).

**Table 3:** Macro and micronutrient status post-sleeve gastrectomy according to GLIM and SGA.

Variable	GLIM			SGA		
	Well-Nourished (*n* = 24)	Malnutrition (*n* = 23)	*p* Value	Well-Nourished (*n* = 24)	Malnutrition (*n* = 23)	*p* Value
Macronutrients						
Calories (Kcal)	779.39 (900.72)	1483.89 (3435.79)	0.157	812.38 (1016.15)	2471.92 (3826.81)	0.115
Carbohydrates (g)	129.65 (608.64)	211.45 (645.61)	0.427	106.48 (148.08)	380.61 (720.28)	0.208
Sugar (g)	53.14 (73.37)	66.89 (61.16)	0.851	43.24 (56.78)	74.87 (63.70)	0.208
Added Sugar (g)	4.78 (38.39)	0.14 (12.50)	0.069	0.70 (32.63)	0.36 (33.01)	0.97
Protein (g)	32.22 (112.36)	98.18 (116.65)	0.135	44.73 (81.07)	120.91 (155.64)	0.098
Fat (g)	14.11 (31.06)	43.03 (43.87)	0.305	15.71 (22.04)	50.25 (56.28)	0.069
Saturated Fat (g)	3.55 (4.98)	7.78 (8.74)	0.135	3.55 (4.89)	7.84 (8.82)	0.135
Monounsaturated Fat (g)	1.76 (7.10)	2.57 (4.32)	0.851	1.35 (3.32)	5.00 (10.00)	0.208
Polyunsaturated Fat (g)	1.2 (4.80)	1.13 (4.17)	0.97	1.04 (3.02)	1.29 (5.96)	0.678
Water (mL)	428.08 (681.76)	198.45 (674.27)	0.624	275.62 (621.18)	331.46 (829.81)	0.678
Micronutrients						
Vitamin A (µg)	59.61 (91.07)	68.01 (193.63)	0.624	72.55 (210.09)	55.09 (69.60)	0.384
Vitamin D (µg)	0.52 (1.40)	0.67 (3.07)	0.678	0.44 (1.88)	0.78 (1.23)	0.792
Vitamin B12 (µg)	0.30 (0.83)	0.89 (0.80)	0.208	0.56 (0.98)	0.46 (0.77)	0.678
Folic acid (µg)	55.88 (62.51)	42.63 (56.86)	0.571	44.36 (40.36)	83.68 (119.76)	0.208
Vitamin E (mg)	0.69 (1.35)	0.56 (1.63)	0.792	0.62 (0.72)	0.88 (2.25)	0.851
Iron (µg)	4.91 (6.66)	15.10 (49.15)	0.521	3.79 (7.02)	27.67 (50.26)	0.069
Zinc (mg)	1.00 (0.60)	1.56 (1.79)	0.624	1.08 (0.80)	1.25 (3.06)	0.624
Copper (mg)	0.14 (0.22)	0.09 (0.28	0.473	0.11 (0.12)	0.18 (0.44)	0.521
Selenium (µg)	11.26 (27.85)	19.43 (79.54)	0.473	17.76 (29.03)	11.26 (27.39)	0.734

Data are presented as median and (IQR).

**Table 4:** Comparison of albumin, NLR, and CRP categories in study groups according to GLIM and SGA.

Variable	GLIM			SGA		
	Well-Nourished *n (% Within Raw)*	Malnutrition *n (% Within Raw)*	*p* Value	Well-Nourished *n (% Within Raw)*	Malnutrition *n (% Within Raw)*	*p* Value
Albumin (g/L)						
Normal	23 (51.1%)	22 (48.9%)	0.975	27 (60%)	18 (40%)	0.093
Low (˂35 g/L)	1 (50.0%)	1 (50.0%)		0	2 (100%)	
NLR						0.124
Normal	24 (51.1%)	23 (48.9%)	0.975	27 (57.4%)	20 (42.6%)	
≥3 or ˂0.7	0	0		0	0	
CRP						
Normal	21 (50%)	21 (50%)	0.672	24 (57.1)	18 (42.9%)	0.903
>3 mg/L	3 (60%)	2 (40%)		3 (60.0)	2 (40%)	

**Table 5:** Prevalence of phenotypic and etiologic GLIM criteria among patients.

	GLIM		
	Well-Nourished (*n* = 24)	Malnutrition (*n* = 23)	*p* Value
Phenotypic criteria			
Weight loss	24 (51.1%)	23 (48.9%)	NA
Low BMI	0 (0%)	1 (100%)	0.302
Reduce Muscle Mass	0 (0.0%)	23 (100)	<0.0001
Etiologic criteria			
Reduce food intake			
25%	3 (50%)	3 (50%)	0.885
50%	11 (47.8%)	12 (52.2%)	
75%	10 (55.6%)	8 (44.4%)	
Diseases/Inflammation	0 (0%)	0 (0%)	NA

BMI = Body Mass Index. Data for categorical variables are expressed as a number and percentage *n* (%). *p*-value ≤ 0.05 is considered statistically significant. NA = not applicable.

**Table 6:** The confusion matrix of actual malnutrition, as confirmed by SGA, and predicted malnutrition by GLIM.

	Actual Malnutrition, as Confirmed by SGA		
Predicted malnutrition by GLIM		Positives	Negatives
	Positives	13	10
	Negatives	9	15

**Table 7:** Main characteristics of false positives and false negatives groups.

	False Positives (*n* = 12)	False Negatives (*n* = 9)	*p* Value
Pre-op weight (kg)	116.42 ± 25.07	126.33 ± 23.83	0.371
Current weight (kg)	80.45 ± 20.96	81.32 ± 19.90	0.924
Current BMI (kg/m^2^)	31.42 ± 6.11	29.29 ± 4.57	0.393
% weight loss (%)	29.23 ± 9.22	33.09 ± 7.35	0.316
Current muscle mass (kg)	46.62 ± 15.62	50.75 ± 11.89	0.516
Current fat mass (kg)	29.83 ± 13.62	27.89 ± 13.96	0.752
Current fat-free mass (kg)	52.66 ± 12.68	53.44 ± 12.64	0.89
Calorie intake (kcal)	795.31 (408.18)	635.26 (326.76)	0.347
CHO intake (g)	107.90 (54.29)	85.42 (44.01)	0.323
Fat intake (g)	20.99 (13.20)	17.80 (12.31)	0.58
Protein (g)	42.91 (29.48)	32.75 (19.42)	0.382

## Data Availability

The data supporting the reported results can be provided by the corresponding author upon reasonable request.
